# The chromosomal genome sequence of the kidney sponge, *Chondrosia reniformis *Nardo, 1847, and its associated microbial metagenome sequences

**DOI:** 10.12688/wellcomeopenres.24166.1

**Published:** 2025-05-29

**Authors:** Lucia Pita, Manuel Maldonado, Vassiliki Koutsouveli, Ana Riesgo, Ute Hentschel, Graeme Oatley, Elizabeth Sinclair, Eerik Aunin, Noah Gettle, Camilla Santos, Michael Paulini, Haoyu Niu, Victoria McKenna, Rebecca O’Brien, Wellcome Sanger Institute Tree of Life Management, Samples and Laboratory Team, Wellcome Sanger Institute Scientific Operations: Sequencing Operations, Wellcome Sanger Institute Tree of Life Core Informatics Team, EBI Aquatic Symbiosis Genomics Data Portal Team, Aquatic Symbiosis Genomics Project Leadership

**Affiliations:** 1 Institute of Marine Sciences – CSIC, Barcelona, Spain; 2 Integrated Marine Ecology group, Institute of Marine Research IIM-CSIC, Vigo, Spain; 3 Department of Marine Ecology, Center for Advanced Studies of Blanes (CEAB-CSIC), Girona, Spain; 4 GEOMAR Helmholtz Centre for Ocean Research Kiel, Kiel, Germany; 5 MNCN-National Museum of Natural Sciences, Department of Biodiversity and Evolutionary Biology, Madrid, Spain; 6 RU Marine Symbioses, GEOMAR Helmholtz Centre for Ocean Research Kiel, Kiel, Germany; 7 Tree of Life, Wellcome Sanger Institute, Hinxton, England, UK

**Keywords:** Chondrosia reniformis, kidney sponge, genome sequence, chromosomal, Chondrillida; microbial metagenome

## Abstract

We present a genome assembly from a specimen of *Chondrosia reniformis* (kidney sponge; Porifera; Demospongiae; Chondrillida; Chondrillidae). The genome sequence has a total length of 117.37 megabases. Most of the assembly (99.98%) is scaffolded into 14 chromosomal pseudomolecules. The mitochondrial genome has also been assembled and is 17.45 kilobases in length. Several symbiotic bacterial genomes were assembled as MAGs. Gene annotation of the host organism assembly on Ensembl identified 17,340 protein-coding genes. The metagenome of the specimen was also assembled and 53 binned bacterial genomes were identified, including 40 high-quality MAGs that were representative of a typical high microbial abundance sponge and included three candiate phyla (Poribacteria, Latescibacteria, Binatota)

## Species taxonomy

Eukaryota; Opisthokonta; Metazoa; Porifera; Demospongiae; Verongimorpha; Chondrillida; Chondrillidae; *Chondrosia*; *Chondrosia reniformis* Nardo, 1847 (NCBI:txid68574)

## Background

*Chondrosia reniformis* (Nardo, 1847), (colloquially called the kidney sponge), is commonly found in the Mediterranean Sea and the Eastern Atlantic Ocean. It inhabits shallow coastal bottoms up to a depth of 50 m ( Lazoski *et al.*, 2001; Wilkinson & Vacelet, 1979), typically growing on walls, overhangs, and cave entrances. The habitus is thickly-encrusting to submassive and lobate, being smooth and slippery to the touch. Colours range from whitish (at the darker sites) to grey and brownish-violet with tabby tinges. In section, the body shows two distinct macroscopic regions. The outer region, a millimetre thick ectosome (also called cortex), is composed of pinacocytes and collagenous fibrils. The interior region, termed the choanosome, contains the mesohyl, with abundant collagen fibrils, a variety of amoebocitic, wandering cells, and choanocyte chambers. The sponge *C. reniformis* lacks a skeleton of siliceous spicules, which has rendered taxonomic assignment at the species level difficult. Instead, the skeletal support is provided by profusion of collagen fibrils that fill the intercellular medium of the mesohyl and that endow the sponge with high body plasticity, including capacity to harden if touched, to detach outgrowths from its body for asexual dispersal, and to creep on the substrata ( Bonasoro *et al.*, 2001). Its abundant production of fibrils of collagen type I and IV has attracted the interest of the biomedical and cosmetic industry and attempts have been made to develop marine cultures of this species ( Gökalp *et al.*, 2019).

Reproduction in this species goes through gonochorism, oviparity and external development, meaning that the individuals have separate sexes (with no recognisable sex dimorphism), females and males respectively releasing eggs and sperm simultaneously into the water column for external fertilisation and further embryo development to a ciliated lecithotrophic larval stage ( Levi & Levi, 1976; Riesgo & Maldonado, 2008). The dispersal ability of the gametes and of the lecithotrophic larvae is assumed to be poor, drifting in currents. Reproduction can also occur asexually via drop-like outgrowths that serve as propagules and result from the high body plasticity of this species ( Di Camillo *et al.*, 2012). The population structure of *C. reniformis* was investigated along the coast of Tunisia using the cytochrome oxidase subunit I (COI) as a marker ( Moussa *et al.*, 2022). The identification of two distinct *C. reniformis* populations is consistent with the Siculo-Tunisian strait that is known to separate the water bodies between the western and eastern Mediterranean.

*C. reniformis* is a high microbial abundance sponge that contains dense and taxonomically diverse symbiotic microbial consortia extracellularly within its mesohyl tissue, amounting up to 70% of the sponge tissue ( Ribes *et al.*, 2012). Fifteen prokaryotic phyla were detected by Illumina amplicon sequencing of the 16S rRNA gene, with the Pseudomonadota (formerly Proteobacteria), Nitrososphaerota (formerly Thaumarchaeota), Acidobacteriota (formerly Acidobacteria), Bacteroidota (formerly Bacteroidetes) and Chloroflexota (formerly Chloroflexi) in decreasing order representing the dominant phyla ( Roveta *et al.*, 2023). Besides, quantitative analysis by real-time PCR confirmed high concentrations of the candidate phylum Poribacteria in this sponge species ( Bayer *et al.*, 2014). On the genus level, *C. reniformis* was dominated by *Pseudomonas*, *Cenarchaeum*, *Acidobacterium*, *Chloroflexus*, *Nitrosococcus* and *Nitrosopumilus*. Three lineages of ammonium-oxidising microorganisms ( *Cenarchaeum symbiosum*, *Nitrosopumilus maritimus*, and *Nitrosococcus* sp.) co-dominated the sponge microbiome, which is consistent with the measurement of high nitrification and high NH _4_^+^ uptake rates in living sponges ( Bayer *et al.*, 2008; Nemoy *et al.*, 2021; Ribes *et al.*, 2012).

*C. reniformis* is of biotechnological interest as a source of several secondary metabolites, collagen, and other molecules, such as the novel protein chondrosin with anti-tumour activity ( Pozzolini *et al.*, 2015; Scarfì *et al.*, 2020). Sustainable exploitation of this sponge species includes aquaculture attempts ( Gökalp *et al.*, 2019). In addition, *C. reniformis* has been used as a bioindicator of seawater pollution with trace elements, such as mercury ( Roveta *et al.*, 2023).

Besides providing insight into the biology of *C. reniformis*, it is hoped that this genome will be useful for comparative studies across a wide range of sponge ecologies, microbiome complexities and functionalities. It will also help to clarify the evolution within demosponges by enlightening the relationships between the subclass Verongimorpha (to which the Order Chondrosida belongs) and the rest of demosponges, and to conduct broader comparisons with cnidarian and bilaterian genomes to reveal cardinal features of animal-microbe symbioses that trace back to the origin of the Metazoa, or earlier.

## Genome sequence report

### Sequencing data

**Figure 1.**
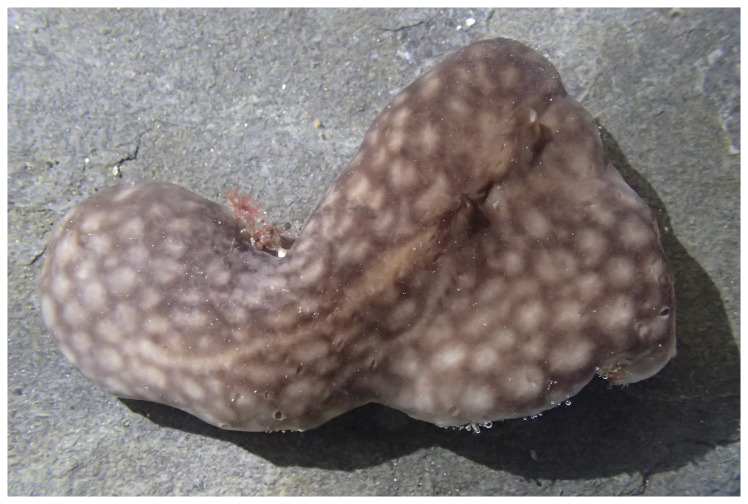
Specimen of *Chondrosia reniformis* (odChoReni1) used for genome sequencing, pictured in the CEAB wet laboratory three hours after its collection and immediately prior to tissue dissection.

The genome of a specimen of *Chondrosia reniformis* ( Figure 1) was sequenced using Pacific Biosciences single-molecule HiFi long reads, generating 10.31 Gb (gigabases) from 0.80 million reads. Based on the estimated genome size, the sequencing data provided approximately 70 coverage of the genome. Chromosome conformation Hi-C data produced 13.26 Gb from 87.83 million reads. RNA sequencing data were also generated and are available in public sequence repositories. Table 1 summarises the specimen and sequencing information.

**Table 1.** Specimen and sequencing data for *Chondrosia reniformis*.

Project information			
**Study title**	Chondrosia reniformis		
**Umbrella BioProject**	PRJEB55903		
**Species**	*Chondrosia reniformis*		
**BioSpecimen**	SAMEA9362004		
**NCBI taxonomy ID**	68574		
Specimen information			
**Technology**	**ToLID**	**BioSample** **accession**	**Organism part**
**PacBio long read sequencing**	odChoReni1	SAMEA9362055	Somatic animal tissue
**Hi-C sequencing**	odChoReni1	SAMEA9362038	Somatic animal tissue
**RNA sequencing**	odChoReni1	SAMEA12929236	Somatic animal tissue
Sequencing information			
**Platform**	**Run accession**	**Read count**	**Base count (Gb)**
**Hi-C Illumina NovaSeq 6000**	ERR10177765	8.78e+07	13.26
**PacBio Sequel IIe**	ERR10224860	8.00e+05	10.31
**RNA Illumina NovaSeq 6000**	ERR10177766	5.77e+07	8.71

## Host assembly statistics

The primary haplotype was assembled, and contigs corresponding to an alternate haplotype were also deposited in INSDC databases. The assembly was improved by manual curation, which corrected 20 misjoins or missing joins and removed 4 haplotypic duplications. These interventions reduced the total assembly length by decreased the scaffold count by 16.67% the scaffold N50 by 3.11%. The final assembly has a total length of 117.37 Mb in 14 scaffolds, and a scaffold N50 of 8.46 Mb ( Table 2).

**Table 2.** Genome assembly data for *Chondrosia reniformis*.

Genome assembly	
Assembly name	odChoReni1.1
Assembly accession	GCA_947172415.1
*Alternate haplotype accession*	*GCA_947172445.1*
Assembly level for primary assembly	chromosome
Span (Mb)	117.37
Number of contigs	42
Number of scaffolds	14
Longest scaffold (Mb)	10.41
Assembly metric	Measure
Contig N50 length	4.71 Mb
Scaffold N50 length	8.46 Mb
Consensus quality (QV)	Primary: 69.8; alternate: 64.2; combined 66.0
*k*-mer completeness	Primary: 69.43%; alternate: 74.54%; combined: 93.73%
BUSCO *	C:71.1%[S:70.4%,D:0.6%],F:12.2%,M:16.8%,n:954
Percentage of assembly mapped to chromosomes	99.98%
Organelles	Mitochondrial genome: 17.45 kb

* BUSCO scores based on the metazoa_odb10 BUSCO set using v5.3.2. C = complete [S = single copy, D = duplicated], F = fragmented, M = missing, n = number of orthologues in comparison. A full set of BUSCO scores is available at https://blobtoolkit.genomehubs.org/view/odChoReni1_1/dataset/odChoReni1_1/busco.

**Figure 2.**
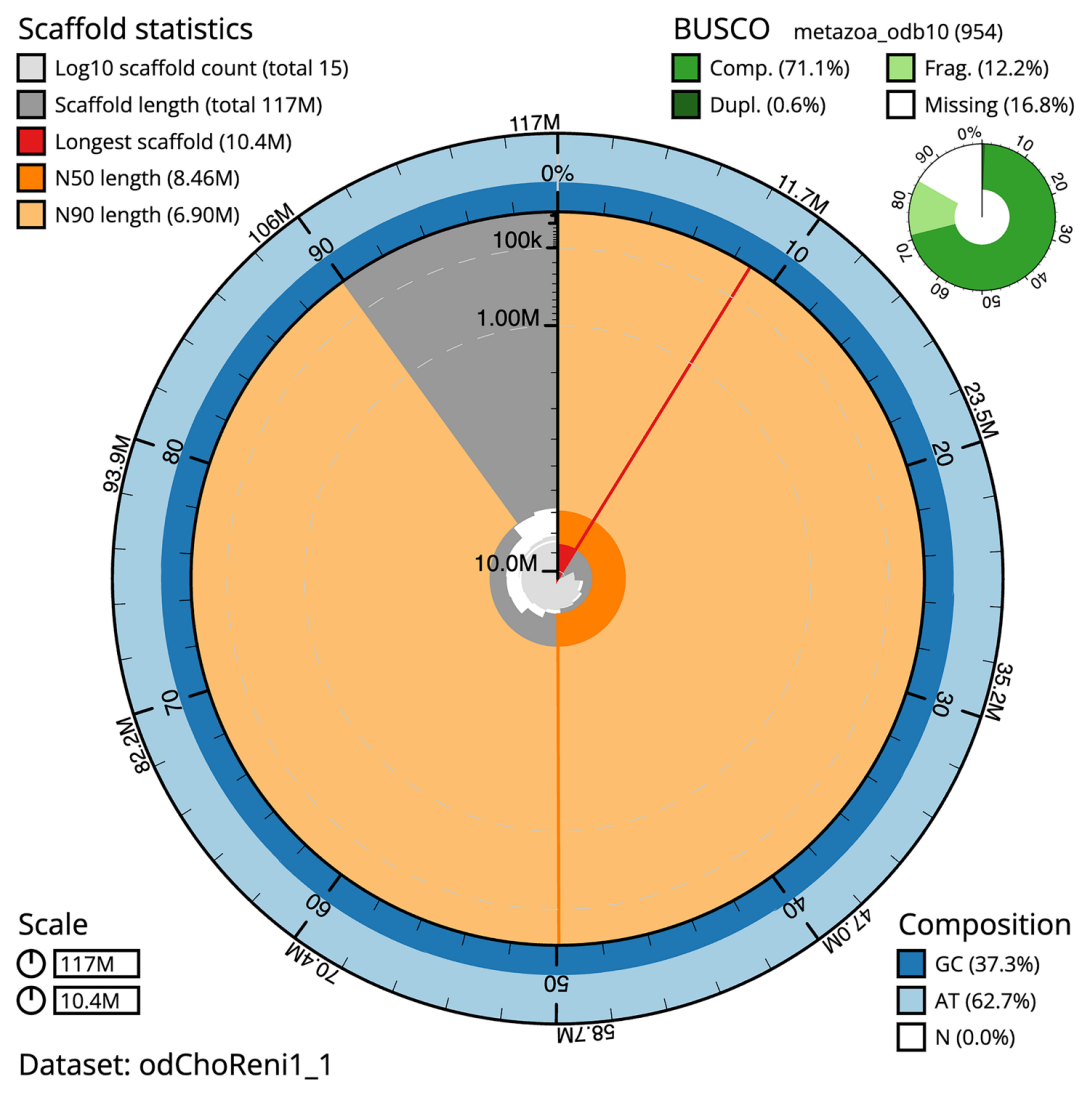
Genome assembly of *Chondrosia reniformis*, odChoReni1.1: metrics. The BlobToolKit Snailplot shows N50 metrics and BUSCO gene completeness. The main plot is divided into 1,000 bins around the circumference with each bin representing 0.1% of the 117,390,217 bp assembly. The distribution of scaffold lengths is shown in dark grey with the plot radius scaled to the longest scaffold present in the assembly (10,413,042 bp, shown in red). Orange and pale-orange arcs show the N50 and N90 scaffold lengths (8,459,200 and 6,903,244 bp), respectively. The pale grey spiral shows the cumulative scaffold count on a log scale with white scale lines showing successive orders of magnitude. The blue and pale-blue area around the outside of the plot shows the distribution of GC, AT and N percentages in the same bins as the inner plot. A summary of complete, fragmented, duplicated and missing BUSCO genes in the metazoa_odb10 set is shown in the top right. An interactive version of this figure is available at https://blobtoolkit.genomehubs.org/view/odChoReni1_1/dataset/odChoReni1_1/snail.

**Figure 3.**
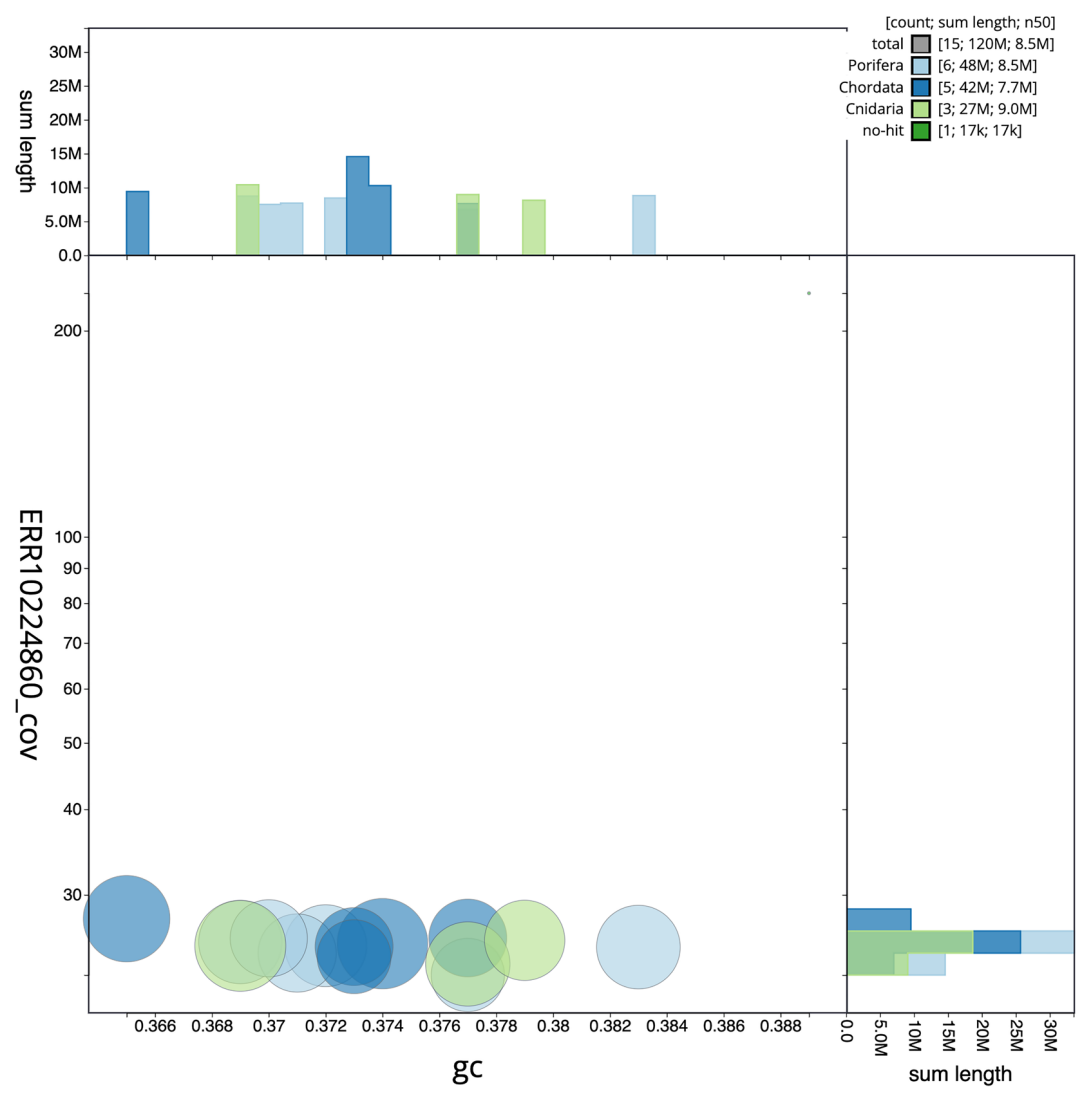
Genome assembly of *Chondrosia reniformis*, odChoReni1.1: BlobToolKit GC-coverage plot. Scaffolds are coloured by phylum. Circles are sized in proportion to scaffold length. Histograms show the distribution of scaffold length sum along each axis. An interactive version of this figure is available at https://blobtoolkit.genomehubs.org/view/odChoReni1_1/dataset/odChoReni1_1/blob.

**Figure 4.**
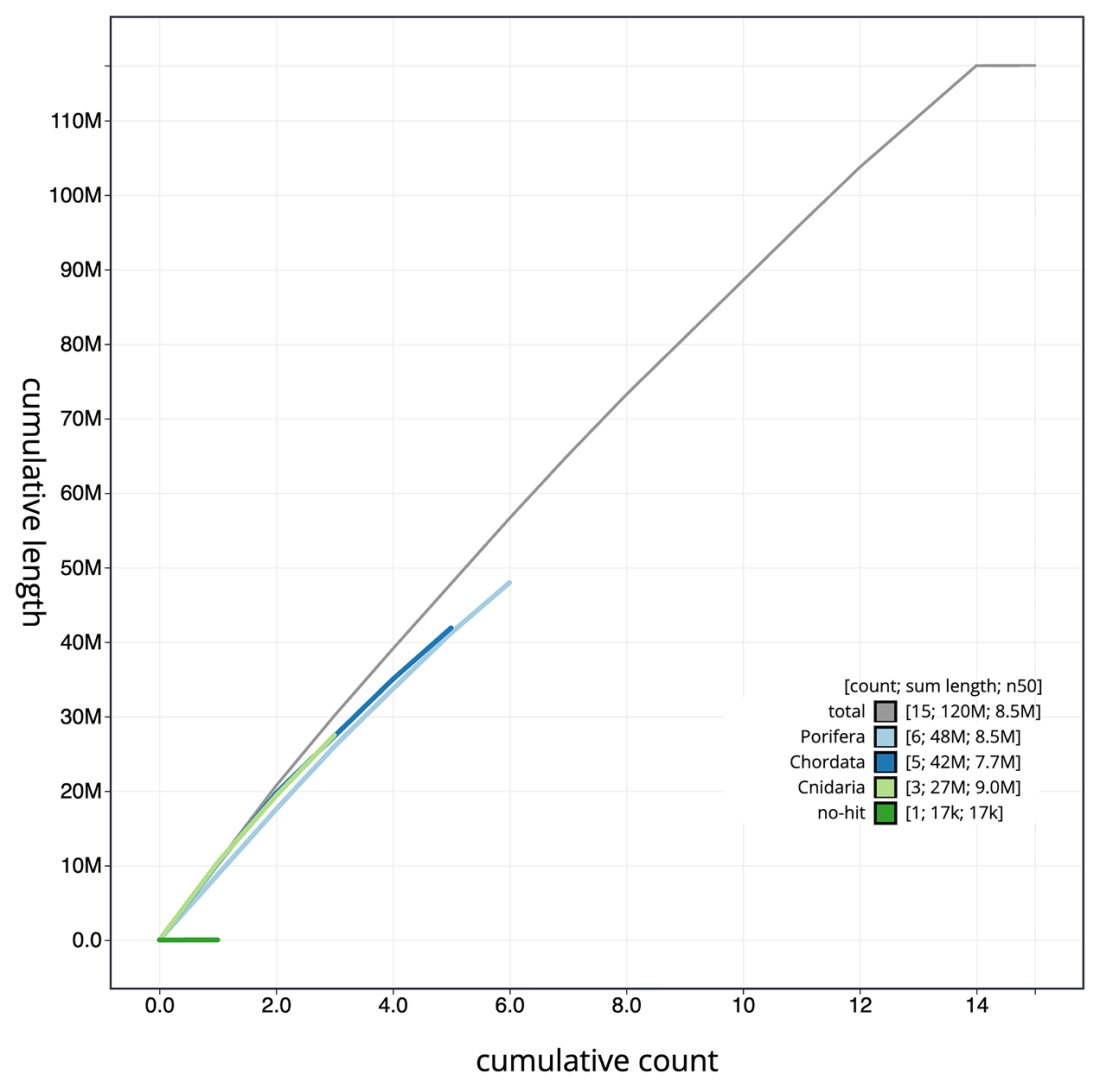
Genome assembly of *Chondrosia reniformis*, odChoReni1.1: BlobToolKit cumulative sequence plot. The grey line shows cumulative length for all scaffolds. Coloured lines show cumulative lengths of scaffolds assigned to each phylum using the buscogenes taxrule. An interactive version of this figure is available at https://blobtoolkit.genomehubs.org/view/odChoReni1_1/dataset/odChoReni1_1/cumulative.

The snail plot in Figure 2 provides a summary of the assembly statistics, indicating the distribution of scaffold lengths and other assembly metrics. Figure 3 shows the distribution of scaffolds by GC proportion and coverage. Figure 4 presents a cumulative assembly plot, with separate curves representing different scaffold subsets assigned to various phyla, illustrating the completeness of the assembly.

**Figure 5.**
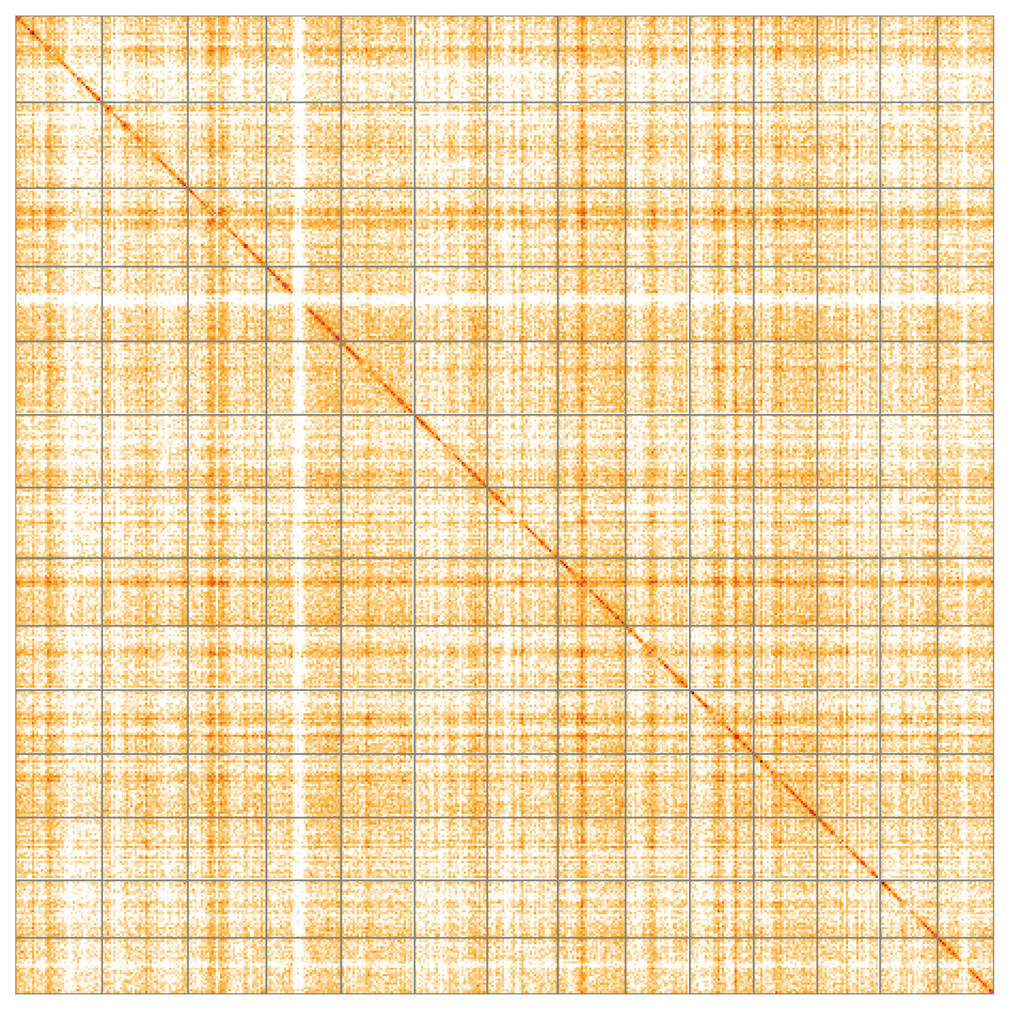
Genome assembly of *Chondrosia reniformis*: Hi-C contact map of the odChoReni1.1 assembly, visualised using HiGlass. Chromosomes are shown in order of size from left to right and top to bottom. An interactive version of this figure may be viewed at https://genome-note-higlass.tol.sanger.ac.uk/l/?d=A60qQ7saS1qCspApxo8K0g.

Most of the assembly sequence (99.98%) was assigned to 14 chromosomal-level scaffolds. These chromosome-level scaffolds, confirmed by Hi-C data, are named according to size ( Figure 5; Table 3).

**Table 3.** Chromosomal pseudomolecules in the genome assembly of *Chondrosia reniformis*, odChoReni1.

INSDC accession	Name	Length (Mb)	GC%
OX359189.1	1	10.41	37
OX359190.1	2	10.29	37.5
OX359191.1	3	9.42	36.5
OX359192.1	4	8.96	37.5
OX359193.1	5	8.8	38.5
OX359194.1	6	8.73	37
OX359195.1	7	8.46	37
OX359196.1	8	8.13	38
OX359197.1	9	7.7	37
OX359198.1	10	7.66	37.5
OX359199.1	11	7.63	37.5
OX359200.1	12	7.52	37
OX359201.1	13	6.9	37.5
OX359202.1	14	6.75	37.5
OX359203.1	MT	0.02	39

The mitochondrial genome was also assembled. This sequence is included as a contig in the multifasta file of the genome submission and as a standalone record in GenBank.

## Host assembly quality metrics

The primary haplotype has a QV of 69.8, and the combined primary and alternate assemblies achieve an estimated QV of 66.0. The host assembly has a BUSCO v5.3.2 completeness of 71.1% (single = 70.4%, duplicated = 0.6%), using the metazoa_odb10 reference set ( *n* = 954).

## Metagenome report

**Figure 6.**
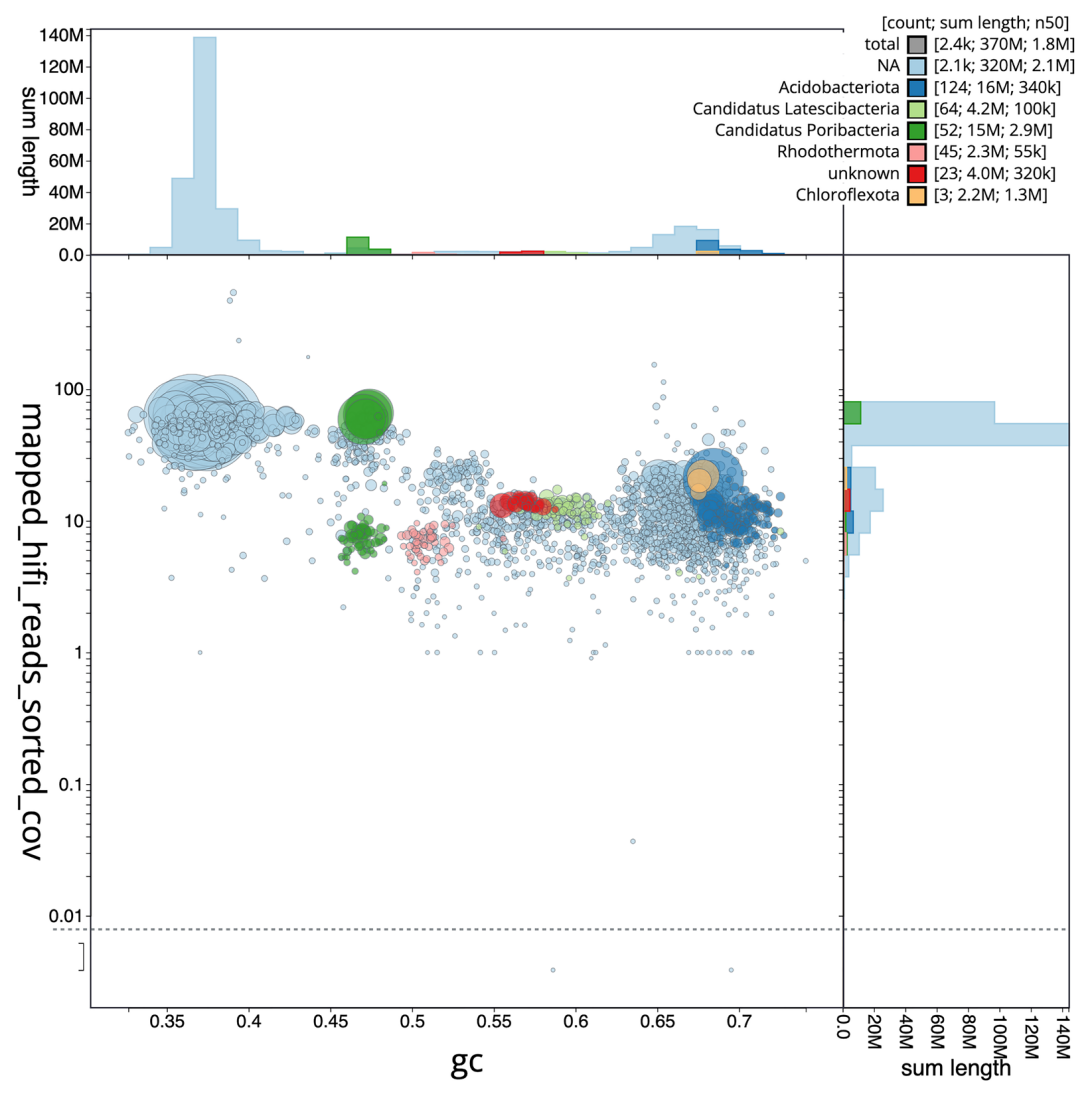
Blob plot of base coverage in mapped against GC proportion for sequences in the *Chondrosia reniformis* metagenome. Binned contigs are coloured by phylum. Circles are sized in proportion to sequence length on a square root scale, ranging from 1,831 to 10,530,910. Histograms show the distribution of sequence length sum along each axis. An interactive version may be viewed here.

**Figure 7.**
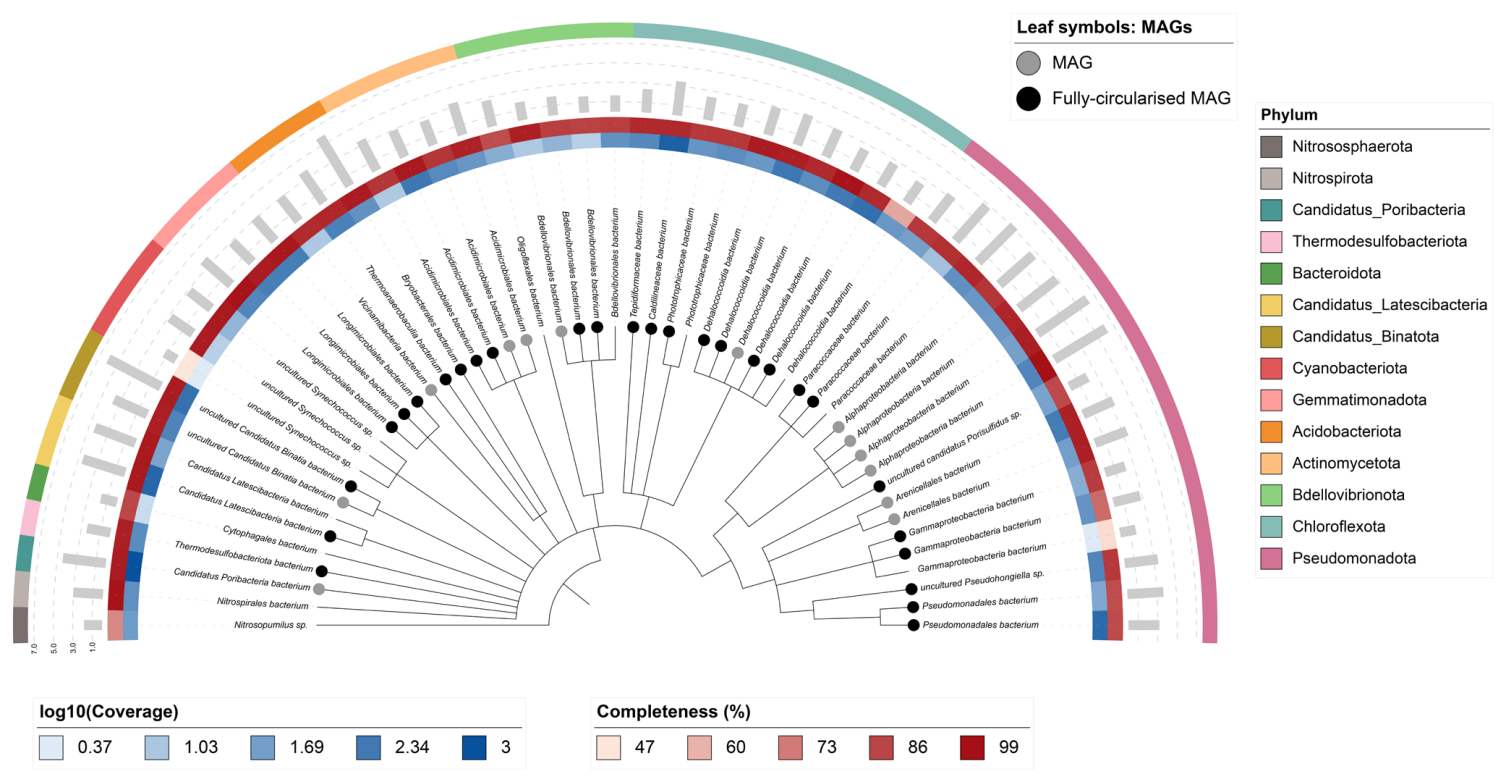
Cladogram showing the taxonomic placement of metagenome bins, constructed using NCBI taxonomic identifiers with *taxonomizr* and annotated in iTOL. Colours indicate phylum-level taxonomy. Additional tracks show sequencing coverage (log _10_), genome size (Mbp), and completeness. Bins that meet the criteria for MAGs are marked with a grey circle and fully circularised MAGs are marked in black.

Fifty-three binned genomes were generated from the metagenome assembly ( Figure 6) of which 40 were classified as high-quality metagenome assembled genomes (MAGs) (see methods). The completeness values for these binned genomes range from approximately 47% to 99% with contamination below 7%. The full set of bins are available on FigShare ( https://doi.org/10.6084/m9.figshare.28777127). A cladogram is shown in Figure 7.

## Host genome annotation report

The *Chondrosia reniformis* genome assembly (GCA_947172415.1) was annotated at the European Bioinformatics Institute (EBI) on Ensembl Rapid Release. The resulting annotation includes 26,350 transcribed mRNAs from 17,340 protein-coding and 784 non-coding genes ( Table 2; https://rapid.ensembl.org/Chondrosia_reniformis_GCA_947172415.1/Info/Index). The average transcript length is 4,621.65. There are 1.45 coding transcripts per gene and 7.20 exons per transcript.

An alternative annotation performed at the WSI using BRAKER3 produced 14,885 genes (coding for 17,554 proteins), with 1.18 coding transcripts per gene and 7.91 exons per transcript. This annotation is provided as UCSC assembly hubs and raw downloads at https://github.com/Aquatic-Symbiosis-Genomics-Project/sponge_annotations/tree/main/results/odChoReni1.

## Methods

### Sample acquisition

A *Chondrosia reniformis* (specimen ID GHC0000189, ToLID odChoReni1) was collected from Blanes, Girona, Spain (latitude 41.67, longitude 2.80) on 2021-02-01. The specimen was taken from the rocky seabed during a SCUBA dive. The specimen was collected and identified by Manuel Maldonado (CEAB-CSIC). Cells were suspended in a solution of artificial seawater containing EDTA.

### Nucleic acid extraction

The workflow for high molecular weight (HMW) DNA extraction at the Wellcome Sanger Institute (WSI) Tree of Life Core Laboratory includes a sequence of procedures: sample preparation and homogenisation, DNA extraction, fragmentation and purification. Detailed protocols are available on protocols.io ( Denton *et al.*, 2023b). The odChoReni1 sample was weighed on dry ice ( Jay *et al.*, 2023). Sponge cells were pelleted by centrifugation (2 minutes at 10,000 rcf), followed by removing the artificial seawater supernatant. The pelleted cells were homogenised using a PowerMasher II tissue disruptor ( Denton *et al.*, 2023a). HMW DNA was extracted using the Manual MagAttract v1 protocol ( Strickland *et al.*, 2023b). DNA was sheared into an average fragment size of 12–20 kb in a Megaruptor 3 system ( Todorovic *et al.*, 2023). Sheared DNA was purified by solid-phase reversible immobilisation, using AMPure PB beads to eliminate shorter fragments and concentrate the DNA ( Strickland *et al.*, 2023a). The concentration of the sheared and purified DNA was assessed using a Nanodrop spectrophotometer and Qubit Fluorometer using the Qubit dsDNA High Sensitivity Assay kit. Fragment size distribution was evaluated by running the sample on the FemtoPulse system.

RNA was extracted from cells of odChoReni1 in the Tree of Life Laboratory at the WSI using the RNA Extraction: Automated MagMax™ *mir*Vana protocol ( do Amaral *et al.*, 2023). The RNA concentration was assessed using a Nanodrop spectrophotometer and a Qubit Fluorometer using the Qubit RNA Broad-Range Assay kit. Analysis of the integrity of the RNA was done using the Agilent RNA 6000 Pico Kit and Eukaryotic Total RNA assay.

### Sequencing

Pacific Biosciences HiFi circular consensus DNA sequencing libraries were constructed according to the manufacturers’ instructions. DNA and RNA sequencing was performed by the Scientific Operations core at the WSI on Pacific Biosciences Sequel II (HiFi) and Illumina NovaSeq 6000 (RNA-Seq) instruments. Hi-C data were also generated from tissue of odChoReni1 using the Arima2 kit and sequenced on the Illumina NovaSeq 6000 instrument.

### Genome assembly, curation and evaluation

***Assembly***

The HiFi reads were assembled using Hifiasm ( Cheng *et al.*, 2021) with the --primary option. Haplotypic duplications were identified and removed using purge_dups ( Guan *et al.*, 2020). The Hi-C reads were mapped to the primary contigs using bwa-mem2 ( Vasimuddin *et al.*, 2019). The contigs were further scaffolded using the provided Hi-C data ( Rao *et al.*, 2014) in YaHS ( Zhou *et al.*, 2023) using the --break option for handling potential misassemblies. The scaffolded assemblies were evaluated using Gfastats ( Formenti *et al.*, 2022), BUSCO ( Manni *et al.*, 2021) and MERQURY.FK ( Rhie *et al.*, 2020).

The mitochondrial genome was assembled using MitoHiFi ( Uliano-Silva *et al.*, 2023), which runs MitoFinder ( Allio *et al.*, 2020) and uses these annotations to select the final mitochondrial contig and to ensure the general quality of the sequence.

***Assembly curation***

The assembly was checked for contamination and corrected using the gEVAL system ( Chow *et al.*, 2016). Manual curation was conducted primarily in PretextView ( Harry, 2022) and HiGlass ( Kerpedjiev *et al.*, 2018), with additional insights provided by JBrowse2 ( Diesh *et al.*, 2023). Scaffolds were visually inspected and corrected as described by Howe *et al*. (2021). Any identified contamination, missed joins, and mis-joins were amended, and duplicate sequences were tagged and removed. The curation process is documented at https://gitlab.com/wtsi-grit/rapid-curation.

***Host assembly quality assessment***

The Merqury.FK tool ( Rhie *et al.*, 2020), run in a Singularity container ( Kurtzer *et al.*, 2017), was used to evaluate assembly quality for the primary and alternate haplotypes using the *k*-mer databases ( *k* = 31) that were computed prior to genome assembly. The analysis outputs included assembly QV scores.

A Hi-C contact map was produced for the final version of the assembly. The Hi-C reads were aligned using bwa-mem2 ( Vasimuddin *et al.*, 2019) and the alignment files were combined using SAMtools ( Danecek *et al.*, 2021). The Hi-C alignments were converted into a contact map using BEDTools ( Quinlan & Hall, 2010) and the Cooler tool suite ( Abdennur & Mirny, 2020). The contact map is visualised in HiGlass ( Kerpedjiev *et al.*, 2018).

The genome was also analysed within the BlobToolKit environment ( Challis *et al.*, 2020) and BUSCO scores ( Manni *et al.*, 2021) were calculated.

***Metagenome assembly***

The metagenome assembly was generated using metaMDBG ( Benoit *et al.*, 2024) and binned using MetaBAT2 ( Kang *et al.*, 2019), MaxBin ( Wu *et al.*, 2014), bin3C ( DeMaere & Darling, 2019), and MetaTOR. The resulting bin sets of each binning algorithm were optimised and refined using MAGScoT ( Rühlemann *et al.*, 2022). PROKKA ( Seemann, 2014) was used to identify tRNAs and rRNAs in each bin, CheckM ( Parks *et al.*, 2015) (checkM_DB release 2015-01-16) was used to assess bin completeness/contamination, and GTDB-TK ( Chaumeil *et al.*, 2022) (GTDB release 214) was used to taxonomically classify bins. Taxonomic replicate bins were identified using dRep ( Olm *et al.*, 2017) with default settings (95% ANI threshold). The final bin set was filtered for bacteria and archaea. All bins were assessed for quality and categorised as metagenome-assembled genomes (MAGs) if they met the following criteria: contamination ≤ 5%, presence of 5S, 16S, and 23S rRNA genes, at least 18 unique tRNAs, and either ≥ 90% completeness or ≥ 50% completeness with fully circularised chromosomes. Bins that did not meet these thresholds, or were identified as taxonomic replicates of MAGs, were retained as ‘binned metagenomes’ provided they had ≥ 50% completeness and ≤ 10% contamination. A cladogram based on NCBI taxonomic assignments was generated using the ‘taxonomizr’ package in R. The tree was visualised and annotated using iTOL ( Letunic & Bork, 2024). Software tool versions and sources are given in Table 4.

**Table 4.** Software tools: versions and sources.

Software tool	Version	Source
BEDTools	2.30.0	https://github.com/arq5x/bedtools2
bin3C	0.3.3	https://github.com/cerebis/bin3C
BLAST	2.14.0	ftp://ftp.ncbi.nlm.nih.gov/blast/executables/blast+/
Blobtools	4.2.1	https://github.com/blobtoolkit/blobtoolkit
BRAKER3	3.0.7	https://github.com/Gaius-Augustus/BRAKER
BUSCO	5.3.2	https://gitlab.com/ezlab/busco
bwa-mem2	2.2.1	https://github.com/bwa-mem2/bwa-mem2
checkM	2015-01-16	https://ecogenomics.github.io/CheckM/
Cooler	0.8.11	https://github.com/open2c/cooler
DIAMOND	2.0.15	https://github.com/bbuchfink/diamond
dRep	3.4.0	https://github.com/MrOlm/drep
fasta_windows	0.2.4	https://github.com/tolkit/fasta_windows
FastK	427104ea91c78c3b8b8b49f1a7d6bbeaa869ba1c	https://github.com/thegenemyers/FASTK
Gfastats	1.3.6	https://github.com/vgl-hub/gfastats
GoaT CLI	0.2.5	https://github.com/genomehubs/goat-cli
GTDB-Tk	1.2.1	https://github.com/Ecogenomics/GTDBTk
Hifiasm	0.16.1	https://github.com/chhylp123/hifiasm
HiGlass	44086069ee7d4d3f6f3f0012569789ec138f42b84aa44357826c0b6753eb28de	https://github.com/higlass/higlass
MAGScoT	1.0.0	https://github.com/ikmb/MAGScoT
MaxBin	2.2.7	https://sourceforge.net/projects/maxbin/
MerquryFK	d00d98157618f4e8d1a9190026b19b471055b22e	https://github.com/thegenemyers/MERQURY.FK
MetaBAT2	2.15-15-gd6ea400	https://bitbucket.org/berkeleylab/metabat
metaMDBG	Pre-release	https://github.com/GaetanBenoitDev/metaMDBG
metaTOR	Pre-release	https://github.com/koszullab/metaTOR
Minimap2	2.24-r1122	https://github.com/lh3/minimap2
MitoHiFi	2	https://github.com/marcelauliano/MitoHiFi
MultiQC	1.14, 1.17, and 1.18	https://github.com/MultiQC/MultiQC
NCBI Datasets	15.12.0	https://github.com/ncbi/datasets
Nextflow		https://github.com/nextflow-io/nextflow
OMARK	version 2023.10	https://github.com/DessimozLab/OMArk
PretextView	0.2	https://github.com/sanger-tol/PretextView
Prokka	1.14.5	https://github.com/tseemann/prokka
purge_dups	1.2.3	https://github.com/dfguan/purge_dups
samtools	1.15.1	https://github.com/samtools/samtools
Seqtk	1.3	https://github.com/lh3/seqtk
Singularity	3.9.0	https://github.com/sylabs/singularity
TETools	1.87	https://github.com/Dfam-consortium/TETools
YaHS	yahs-1.1.91eebc2	https://github.com/c-zhou/yahs

### Genome annotation method

The Ensembl Genebuild annotation system ( Aken *et al.*, 2016) at the EBI was used to generate annotation for the *Chondrosia reniformis* assembly (GCA_947172415.1). Annotation was created primarily through alignment of transcriptomic data to the genome, with gap filling via protein-to-genome alignments of a select set of proteins from UniProt ( Bateman *et al.*, 2023).

For annotation at the WSI, repeats were annotated using TETools 1.87 to softmask the assembly before predicting genes using BRAKER3 ( Stanke *et al.*, 2008). The protein data used was porifera proteins from UniProt (25th August 2023), combined with protein sets from other ASG porifera predicted also with BRAKER3 to bootstrap the set. For transcriptome evidence 45 million semi-randomly sampled ( Stanke *et al.*, 2019) RNASeq spots from SRA were used. The gene set was also checked for completeness and contaminations using Omark ( Nevers *et al.*, 2025).

### Wellcome Sanger Institute – Legal and Governance

The materials that have contributed to this genome note have been supplied by a Tree of Life collaborator. The Wellcome Sanger Institute employs a process whereby due diligence is carried out proportionate to the nature of the materials themselves, and the circumstances under which they have been/are to be collected and provided for use. The purpose of this is to address and mitigate any potential legal and/or ethical implications of receipt and use of the materials as part of the research project, and to ensure that in doing so we align with best practice wherever possible. The overarching areas of consideration are:

•   Ethical review of provenance and sourcing of the material

•   Legality of collection, transfer and use (national and international)

Each transfer of samples is undertaken according to a Research Collaboration Agreement or Material Transfer Agreement entered into by the Tree of Life collaborator, Genome Research Limited (operating as the Wellcome Sanger Institute) and in some circumstances other Tree of Life collaborators.

## Data Availability

European Nucleotide Archive: Chondrosia reniformis. Accession number PRJEB55903; https://identifiers.org/ena.embl/PRJEB55903. The genome sequence is released openly for reuse. The *Chondrosia reniformis* genome sequencing initiative is part of the Aquatic Symbiosis Genomics (ASG) project ( https://www.ebi.ac.uk/ena/browser/view/PRJEB43743). All raw sequence data and the assembly have been deposited in INSDC databases. Raw data and assembly accession identifiers are reported in Table 1 and Table 2.

## Author information

Members of the Wellcome Sanger Institute Tree of Life Management, Samples and Laboratory Team are listed here: https://doi.org/10.5281/zenodo.10066175.

Members of the Wellcome Sanger Institute Scientific Operations: Sequencing Operations are listed here: https://doi.org/10.5281/zenodo.10043364.

Members of the Wellcome Sanger Institute Tree of Life Core Informatics team are listed here: https://doi.org/10.5281/zenodo.10066637.

Members of the European Bioinformatics Institute ASG Data Portal team are listed here: https://doi.org//10.5281/zenodo.10076466.

Members of the Wellcome Sanger Institute/Aquatic Symbiosis Genomics Project Leadership are listed here: https://doi.org/10.5281/zenodo.10184833.
