# Supplementary material for: The chromosomal genome sequence of the kidney sponge, Chondrosia reniformis Nardo, 1847, and its associated microbial metagenome sequences
Source: Wellcome Open Res. 2025 May 29;10:283. [Version 1] doi: 10.12688/wellcomeopenres.24166.1 (PMC12181767; doi:10.12688/wellcomeopenres.24166.1)
Supplement: Peer review history

# Reviewer report: Reviewer response for version 1

**DOI:** 10.21956/wellcomeopenres.26657.r124623

**Published:** 2025-07-22

**Authors:** Joseph B. Kelly

**Affiliations:** 1 University of Konstanz, Konstanz, Germany

The authors present a highly contiguous genome assembly of a host sponge, *Chondrosia reniformis*, and the metagenome-assembled genomes (MAGs) of 53 associated bacterial strains. The work constitutes a valuable dataset given its potential to address basic biological questions concerning genome evolution in the ‘basal’ phylum Porifera and could also be used to further elucidate host-microbiome crosstalk in high microbial abundance sponges. Suggestions for improving the manuscript follow.

 Even though the genome is in 14 scaffolds, the percent of single-copy complete BUSCOs recovered is quite low. Have the authors tried assembling rarefied versions of the raw read dataset to see if the single-copy complete BUSCO score reaches a plateau? It would be informative to also run the BUSCO software on the *in-silico* predicted proteome to see if the low score could be due to the software's gene prediction step.

 More metadata should be included in the 'Sample acquisition' subsection of the 'Methods' section and ideally also in the assembly’s INSDC database pages. The reporting of more environmental parameters that could influence microbiome composition would improve the usefulness of the MAG dataset for other studies. This could include, for example, the depth that the specimen was taken from, notes on the surrounding biota, and the water temperature. Also include a photo of the sample *in-situ* if it is available.

 Please include details regarding RNA library pipeline (kit used, deviations from the manufacturer’s protocol).

 ‘Porifera’ should be capitalized in ‘Genome annotation method’.

 Under the ‘Sequencing data’ subsection of ‘Genome sequence report’, I recommend rewriting ‘70 coverage’ as ‘70x coverage’ for the sake of clarity.

Are sufficient details of methods and materials provided to allow replication by others?

Yes

Is the rationale for creating the dataset(s) clearly described?

Yes

Are the datasets clearly presented in a useable and accessible format?

Partly

Are the protocols appropriate and is the work technically sound?

Yes

Reviewer Expertise:

Bioinformatics, comparative genomics, metagenomics.

I confirm that I have read this submission and believe that I have an appropriate level of expertise to confirm that it is of an acceptable scientific standard.

# Reviewer report: Reviewer response for version 1

**DOI:** 10.21956/wellcomeopenres.26657.r124619

**Published:** 2025-06-20

**Authors:** Matsapume Detcharoen

**Affiliations:** 1 Prince of Songkla University, Hat Yai, Thailand

This manuscript presents a high-quality chromosomal-level genome assembly of *Chondrosia reniformis*, alongside a comprehensive analysis of its associated microbial genomes. It contributes a valuable genomic resource that will benefit researchers in marine biology, evolutionary genomics, and symbiosis research.

 One of the major strengths of the manuscript is the quality of the host genome assembly. The authors achieved a near-complete chromosomal representation. In addition, the authors identified 40 high quality MAGs.

 Only a few areas could be improved to enhance the manuscript's impact, such as a deeper functional analysis of the host genome and phylogenetic analysis of MAGs. Also, the interactive plot of Figure 6 is currently not working.

Are sufficient details of methods and materials provided to allow replication by others?

Yes

Is the rationale for creating the dataset(s) clearly described?

Yes

Are the datasets clearly presented in a useable and accessible format?

Yes

Are the protocols appropriate and is the work technically sound?

Yes

Reviewer Expertise:

Symbiosis, genomics

I confirm that I have read this submission and believe that I have an appropriate level of expertise to confirm that it is of an acceptable scientific standard.

# Reviewer report: Reviewer response for version 1

**DOI:** 10.21956/wellcomeopenres.26657.r124622

**Published:** 2025-06-17

**Authors:** Emily C Giles

**Affiliations:** 1 Biosecurity, Cawthron Institute (Ringgold ID: 5732), Nelson, Nelson, New Zealand

The authors present a genome assembly and annotations for the demosponge Chondrosia reniformes. PacBio HiFi and HiC were used for the assembly. The assembly is 117.37 Mb in length and is almost completely contained in 14 pseudo chromosomes. Approximately 17K proteins were predicted (Ensemble vs Braker). The authors also present 53 binned bacterial genomes, 40 of which are high quality. Overall, these resources will be valuable to the wider scientific community. The authors clearly describe their methods and results. My only comments/ thoughts:

 1. Perhaps the authors could comment on the extremely low GC content recovered for the host sponge and how this relates to the GC content of its microbial community.

 2. Fragmented BUSCOs of the host are a bit high. Could this be due to the difficulty of assembling HMA sponge genomes?

 3. Number of exons per transcript also strikes me as high, is this common for demosponges?

Are sufficient details of methods and materials provided to allow replication by others?

Yes

Is the rationale for creating the dataset(s) clearly described?

Yes

Are the datasets clearly presented in a useable and accessible format?

Yes

Are the protocols appropriate and is the work technically sound?

Yes

Reviewer Expertise:

comparative genomics, population genomics, speciation

I confirm that I have read this submission and believe that I have an appropriate level of expertise to confirm that it is of an acceptable scientific standard.

# Reviewer report: Reviewer response for version 1

**DOI:** 10.21956/wellcomeopenres.26657.r124621

**Published:** 2025-06-12

**Authors:** Jean-Marc Aury

**Affiliations:** 1 Génomique Métabolique, French Alternative Energies and Atomic Energy Commission Genoscope National Sequencing Center, Évry-Courcouronnes, Île-de-France, France

I read the manuscript describing the genome assembly of the sponge *Chondrosia reniformis* and its associated microbiome with great interest. The resulting assembly appears to be of high quality, which is noteworthy given that sponges are challenging organisms to work with due to their high bacterial content.

 My major concern relates to the assembly process, for which too few details are provided to allow scientific reproducibility. Specifically, assembling the entire PacBio dataset should normally result in a total assembly size greater than the expected genome size of the sponge, due to the presence of bacterial sequences. However, this is not reported here. Similarly, the authors should have removed bacterial contigs from the host assembly, but they do not explain how this was done or provide statistics regarding non-host contigs/scaffolds.

 Additionally, it is unclear whether the authors assembled the full PacBio dataset to reconstruct MAGs. If so, they should have filtered out host-derived contigs, but this is not mentioned.

 Please provide more information on how the presence of multiple organisms was handled during the generation of both the host assembly and the metagenome assembly.

 Finally, the manuscript reports the generation of 40 high-quality MAGs, but upon checking the ENA repository, I could only find 11. Could the authors clarify this discrepancy?

 One minor point: in the abstract, the sentence “Several symbiotic bacterial genomes were assembled as MAGs.” is redundant with the following sentence and should be removed.

 Congratulations to the authors on assembling such a complex sample.

Are sufficient details of methods and materials provided to allow replication by others?

No

Is the rationale for creating the dataset(s) clearly described?

Yes

Are the datasets clearly presented in a useable and accessible format?

Yes

Are the protocols appropriate and is the work technically sound?

Yes

Reviewer Expertise:

genome assembly, comparative genomics

I confirm that I have read this submission and believe that I have an appropriate level of expertise to confirm that it is of an acceptable scientific standard, however I have significant reservations, as outlined above.
